# Intramuscular vaccination against SARS-CoV-2 transiently induces neutralizing IgG rather than IgA in the saliva

**DOI:** 10.3389/fimmu.2024.1330864

**Published:** 2024-02-05

**Authors:** Stephan Winklmeier, Heike Rübsamen, Ceren Özdemir, Paul R. Wratil, Gaia Lupoli, Marcel Stern, Celine Schneider, Katharina Eisenhut, Samantha Ho, Hoi Kiu Wong, Damla Taskin, Marvin Petry, Michael Weigand, Peter Eichhorn, Bärbel U. Foesel, Simone Mader, Oliver T. Keppler, Tania Kümpfel, Edgar Meinl

**Affiliations:** ^1^ Institute of Clinical Neuroimmunology, University Hospital, Ludwig-Maximilians-Universität München, Munich, Germany; ^2^ Biomedical Center (BMC), Medical Faculty, Ludwig-Maximilians-Universität München, Martinsried, Germany; ^3^ Max von Pettenkofer Institute & Gene Center, Virology, Ludwig-Maximilians-Universität München, Munich, Germany; ^4^ German Center for Infection Research (DZIF), Partner Site Munich, Munich, Germany; ^5^ Institute of Laboratory Medicine, University Hospital, Ludwig-Maximilians-Universität München, Munich, Germany; ^6^ Institute of Epidemiology, Helmholtz Munich, Neuherberg, Germany

**Keywords:** viral neutralization, saliva, COVID, vaccination, IgG, IgA

## Abstract

The mucosal immunity is crucial for restricting SARS-CoV-2 at its entry site. Intramuscularly applied vaccines against SARS-CoV-2 stimulate high levels of neutralizing Abs in serum, but the impact of these intramuscular vaccinations on features of mucosal immunity is less clear. Here, we analyzed kinetic and functional properties of anti-SARS-CoV-2 Abs in the saliva after vaccination with BNT162b2. We analyzed a total of 24 healthy donors longitudinally for up to 16 months. We found that specific IgG appeared in the saliva after the second vaccination, declined thereafter and reappeared after the third vaccination. Adjusting serum and saliva for the same IgG concentration revealed a strong correlation between the reactivity in these two compartments. Reactivity to VoCs correlated strongly as seen by ELISAs against RBD variants and by live-virus neutralizing assays against replication-competent viruses. For further functional analysis, we purified IgG and IgA from serum and saliva. In vaccinated donors we found neutralizing activity towards authentic virus in the IgG, but not in the IgA fraction of the saliva. In contrast, IgA with neutralizing activity appeared in the saliva only after breakthrough infection. In serum, we found neutralizing activity in both the IgA and IgG fractions. Together, we show that intramuscular mRNA vaccination transiently induces a mucosal immunity that is mediated by IgG and thus differs from the mucosal immunity after infection. Waning of specific mucosal IgG might be linked to susceptibility for breakthrough infection.

## 1 Introduction

Since the entry site of SARS-CoV-2 is the upper respiratory tract, the mucosal immunity in this area is crucial for fighting the infection in the first place (1, 2). Analysis of the humoral immunity at mucosal sites has historically been focused on IgA, but growing evidence points to a contribution of IgG and IgM to mucosal immunity (3).

In COVID-19 patients, mucosal IgA is induced and highly efficient in neutralizing SARS-CoV-2 (4–6). This IgA is produced locally at the mucosal sites and then released as secretory (s)IgA, which is dimeric; containing the secretory component and the joining (J)-chain. The J-chain serves as a ligand for the polymeric immunoglobulin receptor (pIgR), which is an IgA transporter expressed by mucosal epithelial cells (3, 7). In contrast, IgA in human blood is monomeric, origins from different sites and affects the immunopathology in different tissues including the brain (8, 9). IgA present in blood is not transported into secretions in the mucosa via passive transudation (10).

In contrast to IgA, circulating IgG is transported across mucosal barriers (10) and the neonatal FcR has been identified to be crucial for this process (11, 12). This mucosal IgG can neutralize their antigen, but can also scavenge luminal antigen for further recognition by the immune system (11, 12).

Intramuscular vaccinations against SARS-CoV-2 stimulate a potent systemic immunity (Ig, memory B cells and T cells) (13). These vaccinations transiently protect from infection and can efficiently prevent serious illness, but breakthrough infections occur quite frequently a few months after the last vaccination. Reasons for these breakthrough infections are reduced cross-reactivity against newly emerging variants of concern (VoCs) and waning immunity in particular at the mucosal sites (2).

While the systemic IgG response after vaccination has been studied in great detail, aspects of mucosal immunity after intramuscular vaccination against SARS-CoV-2 have been analyzed less extensively and yielded partially contradictory results. For example, BNT vaccine was reported to induce IgG and little or no specific IgA in the saliva with unclear neutralizing activity (14), while others reported the induction of both SARS-CoV-2-specific IgG and mucosal IgA after vaccination (15–18). Yet another study described systemic, but not mucosal SARS-CoV-2-specific Ig after vaccination (19). Thus, although there is evidence that vaccinations induce anti-SARS-CoV-2 immunity in the saliva, detailed functional features of the mucosal IgG and IgA after vaccination are still unclear.

Here, we performed a longitudinal study analyzing serum and saliva responses from 24 healthy donors after one to three vaccinations over a period of up to 16 months. We set out to compare the functional activity of SARS-CoV-2-specific IgG and IgA in the saliva in relation to serum. To this end, we purified IgG and IgA from both saliva and serum, analyzed their neutralizing activity against SARS-CoV-2 in whole virus assays and determined the reactivity against VoCs. We compared this functional activity of IgG and IgA fractions from vaccinated people and those who underwent a breakthrough infection. Thereby, we found that vaccination induces a mucosal immunity that is distinct from the mucosal immunity after infection.

## 2 Materials and methods

### 2.1 Study participants and sample processing

We analyzed the humoral response of 24 healthy donors (62.5% female; mean age = 37.2 ± 12.6 years) before and after one to three intramuscular injections with the BNT162b2 COVID-19 vaccine from Pfizer–BioNTech. None of the donors had severe side effects of the vaccination. Samples were taken for up to the following ten different time points: 1 = before first vaccination; 2 = two weeks after first vaccination; 3 = before second vaccination; 4 = two weeks after second vaccination; 5 = six weeks after second vaccination; 6 = three months after second vaccination; 7 = six months after second vaccination; 8 = two weeks after third vaccination; 9 = three months after third vaccination; 10 = six months after third vaccination. The time points 4, 8, 9 and 10 were confirmed to be negative for antibodies against the SARS-CoV-2 nucleocapsid protein. Further, we included 12 participants (50.0% female, mean age = 30.1 ± 10.5 years) with three COVID-19 vaccinations and an additional SARS-CoV-2 breakthrough infection, which was confirmed by SARS-CoV-2-specific PCR. These subjects had a mild disease course during the late Delta and early Omicron period and were not hospitalized. Serum was stored at -80°C. Saliva was collected in 50 ml falcon tubes, centrifuged for 10 min at 4000 g and stored at -80°C.

The study was approved by the ethical committee of the medical faculty of the LMU Munich. Written informed consent was obtained from each donor prior to their inclusion in the study.

### 2.2 Detection of SARS-CoV-2 spike protein-specific IgG and IgA

SARS-CoV-2-specific IgG and IgA were detected in sera and specific IgG in saliva using EUROIMMUN anti-SARS-CoV-2 ELISA kits coated with the S1 domain of the SARS-CoV-2 spike protein (EUROIMMUN, Lübeck, Germany). Serum was diluted starting with 1:101 in sample buffer as indicated in the manufacturer’s protocol, and saliva was applied with a 1:2 dilution. Samples were serially diluted with the factor two in order to obtain optical density (OD) values below 2.5 and the applied dilution factor was considered accordingly in the calculation. EUROIMMUN uses a ratio-based analysis for test evaluation and recommends interpreting the results as follows: negative (ratio <0.8), borderline (ratio ≥0.8 to <1.1), positive (ratio ≥1.1).

For determining SARS-CoV-2-specific secretory IgA levels in saliva, an in-house RBD ELISA was used as described in (14). Briefly, half-area ELISA plates were coated with 50 µL of SARS-CoV-2 wild type (WT) RBD (2 µg/mL; PX-COV-P046, ProteoGenix, Schiltigheim, France) or bovine serum albumin (BSA, 2 µg/mL, Sigma-Aldrich) overnight at 4°C. The plates were then blocked for 1 h at room temperature with 100 µL of blocking buffer (3% milk in PBS containing 0.05% Tween 20 (PBST)). Saliva samples were diluted 1:5 in sample buffer (1% milk in 0,1% PBST), 50 µL was added per well and incubated at room temperature (RT) for 2 h. Abs were then detected with 50 µL of anti-human IgA alkaline phosphatase (AP)-conjugated secondary antibody (1:16000, A18790, Thermo Fisher Scientific, Waltham, MA, USA) and 50 µL of 1-Step™ PNPP solution (37621, Thermo Fisher Scientific) as the substrate. The reaction was stopped by adding 25 µL of 2 N sodium hydroxide. The OD of the chromogenic reaction was measured at 405 nm, and the plate background at 540 nm was subtracted.

### 2.3 Detection of SARS-CoV-2 nucleocapsid protein-specific Abs

SARS-CoV-2 nucleocapsid antibodies were measured in sera using the Elecsys Anti-SARS-CoV-2 sandwich assay on a Cobas e801 module (Roche Diagnostics, Mannheim, Germany) and the analysis was conducted according to the manufacturer’s instructions. The assay employs a SARS-CoV-2 specific recombinant antigen representing the nucleocapsid protein. The electrochemiluminescent signal obtained was compared to the cut-off signal value previously generated by two calibrators. The results were expressed as cut-off index, negative COI <1.0 or positive COI ≥1.0, for anti-SARS-CoV-2 total antibodies.

### 2.4 Cross-reactivity to RBD of variants of concern

The cross-reactivities of Abs recognizing SARS-CoV-2 WT to RBDs of VoCs Alpha/B.1.1.7 (mutation N501Y), Beta/B.1.351 (mutations K417N, E484K, N501Y), Gamma/P.1 lineage (mutations K417T, E484K, N501Y), also called B.1.1.248, Delta/B.1.617.2 (mutations L452R, T478K), and Omicron/B.1.1.529 BA.1 (mutations A67V, Δ69-70, T95I, G142D, Δ143-145, Δ211-212, ins214EPE, G339D, S371L, S373P, S375F, K417N, N440K, G446S, S477N, T478K, E484A, Q493K, G496S, Q498R, N501Y, Y505H, T547K, D614G, H655Y, N679K, P681H, N764K, D796Y, N856K, Q954H, N969K, L981F) (20) were determined by ELISA. Half-area ELISA plates were coated with 50 µL of 2 µg/mL RBD WT (PX-COV-P046, ProteoGenix), RBD Alpha (PX-COV-P052, ProteoGenix), RBD Beta (PX-COV-P053, ProteoGenix), RBD Gamma (PX-COV-P054, ProteoGenix), Delta (PX-COV-P061, ProteoGenix), Omicron (PX-COV-P074, ProteoGenix), or BSA (2 µg/mL; Sigma-Aldrich) overnight at 4°C. The subsequent procedure was as described in (Winklmeier et al., 2022). Briefly, the plates were blocked for 2 h at 37°C with 100 µL of blocking buffer (3% milk in PBST). Serum (1:100) or saliva (1:2) were diluted in PBST containing 1% BSA, 50 µL was added per well and incubated at RT for 2 h. Abs were then detected with 50 µL of anti-human IgG horseradish peroxidase (1:5000, 109-036-003, Jackson ImmunoResearch, West Grove, PA, USA) and 50 µL of tetramethylbenzidin (TMB, Sigma-Aldrich) as the substrate. The reaction was stopped by adding 25 µL of 1 M sulfuric acid. The OD of the chromogenic reaction was measured at 450 nm, and the plate background at 540 nm was subtracted.

### 2.5 Purification of IgG and IgA

IgG and IgA were purified from blood (plasma/serum) or saliva using IgG NAb™ protein G spin kit (89979, Thermo Fisher) and the CaptureSelect™ IgA affinity matrix (194288005, Thermo Fisher). Samples were collected 2-4 weeks after the third vaccination. Briefly, for IgG purification, columns were equilibrated by adding 2 mL of binding buffer and centrifuged for 1 minute. Filtered samples were loaded subsequently and incubated at RT with end-over-end mixing for 10 minutes. Columns were washed three times by adding 2 mL of binding buffer and centrifuged for 1 minute. Neutralization buffer (50 µl) was added to a 15 mL collection tube, the spin column placed into that tube, 500 µl elution buffer added to the column and centrifuged for 1 minute. Further elution fractions were obtained by repeating these steps to a total of five times. For IgA purification, the columns were packed with the resin and equilibrated three times with 3 ml of PBS. Filtered samples were loaded subsequently and incubated at RT with end-over-end mixing for 30 minutes. Columns were washed four times by adding 3 mL of PBS and centrifugation for 1 minute. Neutralization buffer (50 µl) was added to a 15 mL collection tube, the column placed into that tube, 500 µl of 0.1M glycine at pH 3 added to the column for elution and centrifuged for 1 minute. Further elution fractions were obtained by repeating these steps to a total of five times. We have analyzed the purified IgG and IgA fractions from plasma and saliva by a non-reducing SDS-PAGE gel. This shows that the purified IgA from the saliva is dimeric while the IgA from the plasma is largely monomeric (**Supplementary Figure 1**).

All fractions from IgG or IgA isolations of each donor and sample were combined, concentrated and buffer-exchanged in PBS with Amicon® Ultra 10K devices (10,000 NMWL, UFC801024, Merck Millipore, Burlington, MA, United States), and the total IgG concentration was measured by human IgG and IgA ELISA development kits (3850-1AD-6, 3860-1AD-6, Mabtech, Nacka Strand, Sweden). Depending on the available amount of material, concentration of 0.3 – 1.0 mg/ml was used for the neutralization assay.

### 2.6 Live virus neutralization assay

The neutralizing activity of sera, plasma, saliva purified IgG and IgA was analyzed using a live virus neutralization assay, as described in (21, 22). Briefly, CaCo-2 cells in cell culture medium (Dulbecco’s Modified Eagle’s Medium containing 2% fetal bovine serum) were challenged for 2 h with clinical isolates of different SARS-CoV-2 variants (EU1/B.1.177, Alpha/B.1.1.7, Beta/B.1.351, Gamma/P.1/B.1.1.28.1, Delta/B.1.617.2, Omicron/B.1.1.529 sublineage BA.1) previously obtained from nasopharyngeal swabs of COVID-19 patients (23, 24). Subsequently, cell culture medium was exchanged, and three days post infection supernatants were passaged on Vero-E6 cells. After three additional days, cell culture supernatants were harvested and stored at -80°C. Virus stocks were characterized by rRT-PCR. A volume of each stock, which results in a 90% cytopathic effect three days post infection, was incubated for 2 h with the neutralizing samples at different dilutions. Subsequently, 10 µL of the virus-sample mixtures were added to 20 µL MDA-MB-231 cells overexpressing the human angiotensin-converting enzyme 2 receptor (hACE2) cultured in 384-well plates (7,500 cells/well). Three days post infection, 10 µL of CellTiter-Glo 2.0 reagent (Promega, Madison, WI, USA) were added to each well and the luminescence was recorded (0.5 s integration time, no filter). Half-maximal inhibitory concentrations (IC_50_) for inhibiting virus-mediated cell death were computed in Prism 9 (GraphPad Software, Boston, MA, USA) via normalized sigmoidal dose-response curve approximation with variable slopes.

### 2.7 Quantification and statistical analysis

Statistical analyses were performed using GraphPad Prism 7.01 and 9.0.2 and are indicated in detail in each figure legend.

## 3 Results

### 3.1 Vaccination transiently induces SARS-CoV-2-specific IgG in the saliva

**Figure 1:**
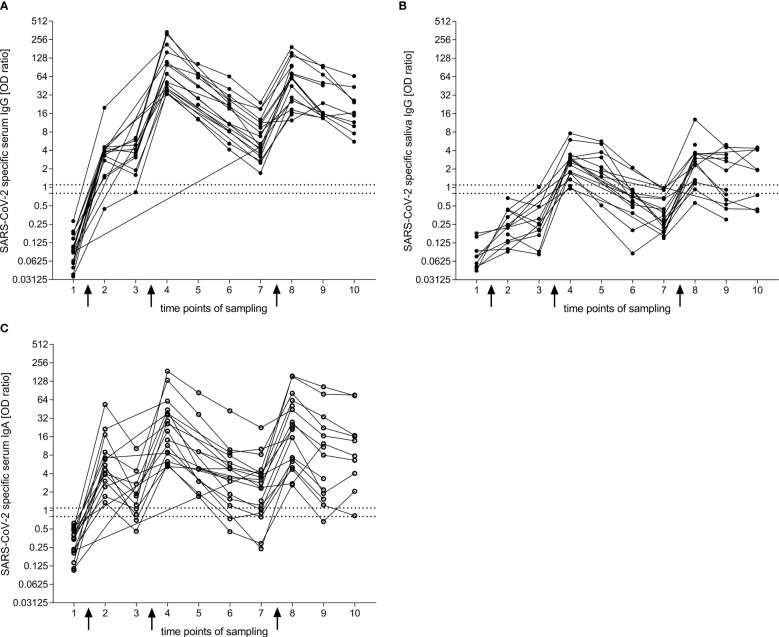
IgG and IgA response to the S1 domain of the SARS-CoV-2 spike protein after BNT vaccination obtained by ELISA. **(A, B)** Longitudinal reactivity of specific IgG in serum **(A)** and saliva **(B)**. **(C)** Longitudinal reactivity of specific IgA in serum. Arrows indicate vaccination time points one, two and three. Connection between dots refer to the response within one donor. Time points of sampling: 1 = before first vaccination (n**_A_** = 17, n**_B_** = 9, n**_C_** = 17); 2 = two weeks after first vaccination (n**_A_** = 14, n**_B_** = 14, n**_C_** = 14); 3 = before second vaccination (n**_A_** = 10, n**_B_** = 10, n**_C_** = 10); 4 = two weeks after second vaccination (n**_A_** = 17, n**_B_** = 16, n**_C_** = 17); 5 = six weeks after second vaccination (n**_A_** = 9, n**_B_** = 9, n**_C_** = 9); 6 = three months after second vaccination (n**_A_** = 14, n**_B_** = 13, n**_C_** = 14); 7 = six months after second vaccination (n**_A_** = 21, n**_B_** = 18, n**_C_** = 21); 8 = two weeks after third vaccination (n**_A_** = 19, n**_B_** = 17, n**_C_** = 19); 9 = three months after third vaccination (n**_A_** = 14, n**_B_** = 12, n**_C_** = 14); 10 = six months after third vaccination (n**_A_** = 10, n**_B_** = 8, n**_C_** = 10). The area between the two horizontal dotted lines in **(A, C)** was considered to represent the borderline zone of reactivity (EUROIMMUN). For comparison, we included these lines for serum also in the graph of saliva IgG. Closed dots represent IgG and open dots IgA.

In our longitudinal study, we analyzed the level of SARS-CoV-2-specific IgG and IgA in serum and saliva from 24 vaccinated healthy donors for a time period of over one year with ELISA (**Figure 1**). A baseline sample was obtained shortly before the first dose of BNT vaccination and the collection continued until six months after the third injection of vaccination. In total, up to ten time points were sampled (mean = 6.0 ± 2.8 time points) for up to 16 months (mean = 9.5 ± 5.2 months). The samples obtained at time points 4, 8, 9 and 10 were confirmed to be negative for antibodies against the SARS-CoV-2 nucleocapsid protein in order to exclude a previous infection. We only included samples from donors who were negative at time point 4 and tested the reactivity at time points 4, 8, 9, and 10. The reactivity against the nucleocapsid protein typically persists for a longer period, so a negative anti-nucleocapsid protein antibody test at time point 4 excludes a previous infection against SARS-CoV-2, since time points 1-3 were only approximately 6 weeks before time point 4 (about two weeks after the second vaccination).

Two weeks after the first dose of vaccination, over 90% of donors developed SARS-CoV-2-specific IgG in their serum (**Figure 1A**). This increased further to 100% two weeks after the second dose. The specific IgG response dropped over the observed period of six months after the second vaccination but stayed positive for all participants. Two weeks after the third vaccination, the specific IgG titers reached a similar level as after the second dose, and decreased more slowly compared with the course of six months after the second vaccination.

In the saliva, the SARS-CoV-2-specific IgG increased more slowly than in the serum and showed a clearly positive response only after the second vaccination (**Figure 1B**). The specific reactivity was transient and dropped faster in saliva compared to serum. After the third vaccination, the specific IgG levels could reach a similar response as seen after the second dose.

After the second vaccination, the proportion of participants with SARS-CoV-2-specific IgA in serum declined from 100% (after 2 weeks) to 71.4% (after 6 months, p = 0.0010, Dunn’s multiple comparisons test) (**Figures 1A, C**). After the third vaccination, SARS-CoV-2-specific IgA in serum was observed in all patients. The level of the serum IgA was similar as seen after the second vaccination, but declined slower; 90% of participants remained positive after 6 months.

For the analysis of specific IgA in the saliva, we could not obtain a clear signal with the EUROIMMUN ELISA kit due to a high background (**Supplementary Figure 2A**). Therefore, we developed an in-house ELISA following a previous publication (14). Also with this test, we did not observe a significant increase of specific IgA after any of the three doses of vaccinations (**Supplementary Figure 2B**). Further, we tested the saliva of donors who had a breakthrough infection with COVID-19 after the third vaccination and were able to detect a specific IgA response compared to baseline saliva (**Supplementary Figure 2C**).

**Figure 2:**
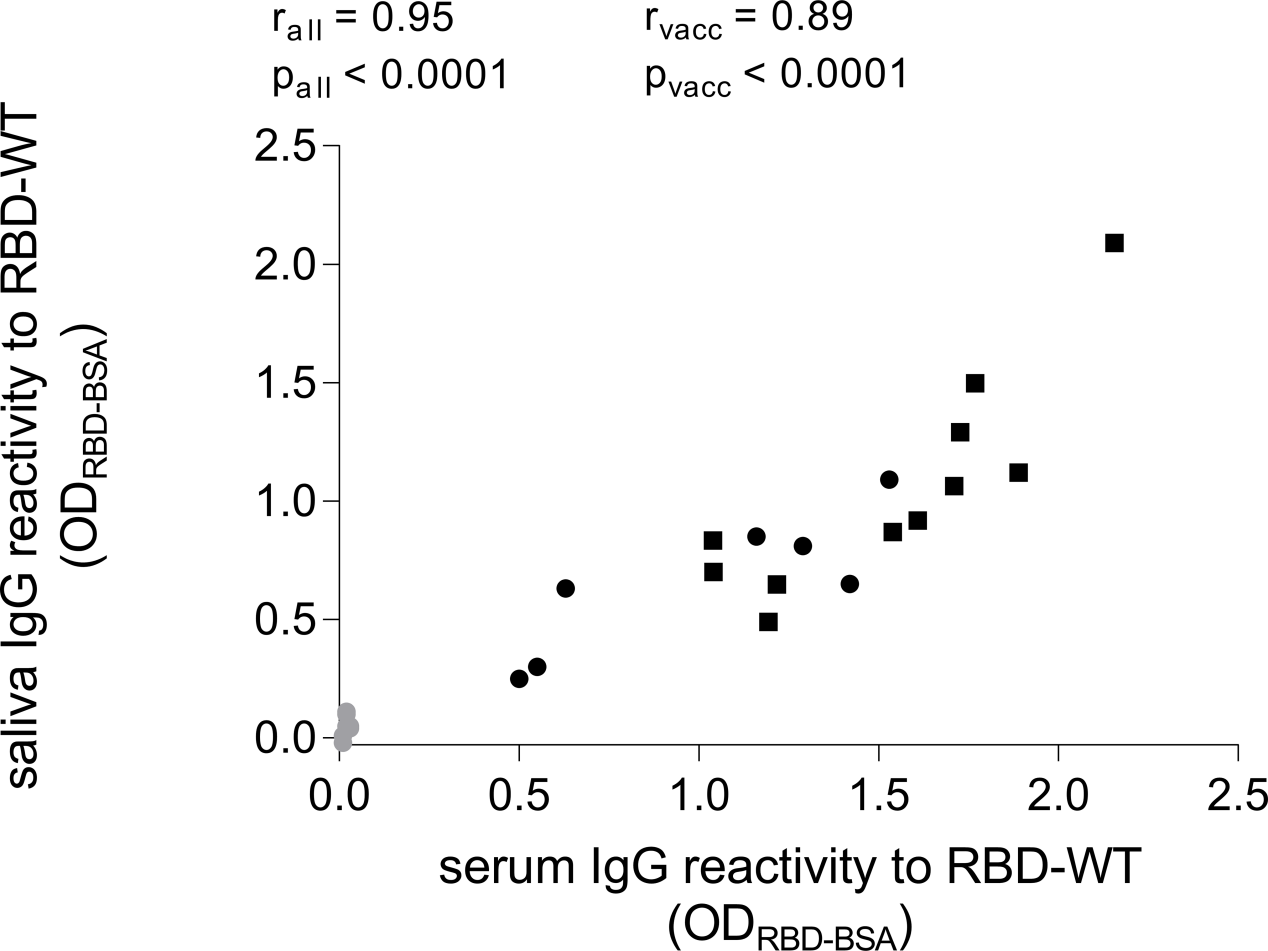
Comparison of SARS-CoV-2-specific IgG levels in serum and saliva. Serum samples were diluted to the same total IgG levels of their corresponding saliva sample and then tested in parallel in an ELISA coated with RBD of SARS-CoV-2 WT. Grey dots represent baseline values (n = 7). Black dots represent time point of two weeks after second vaccination (n = 7). Black rectangles represent time point of two weeks after third vaccination (n = 11). IgG levels in serum did correlate with the IgG levels in saliva (Spearman correlation, for all values r_all_ = 0.95, for time points only two weeks after second and third vaccination r_vacc_ = 0.89).

To compare the association between SARS-CoV-2-specific IgG in serum and saliva, we determined the total IgG concentration in each sample (mean of serum IgG = 7.8 ± 1.8 mg/ml, mean of saliva IgG = 14.7 ± 13.2 µg/ml). Subsequently, we diluted the serum to the concentration of the saliva and compared the reactivity to RBD WT by the two samples side-by-side in an ELISA. Hereby, we observed a significant correlation between the SARS-CoV-2-specific IgG levels in serum and saliva (**Figure 2**, r = 0.95, p < 0.0001).

### 3.2 Cross-reactivity to VoCs closely correlate in serum and saliva

**Figure 3:**
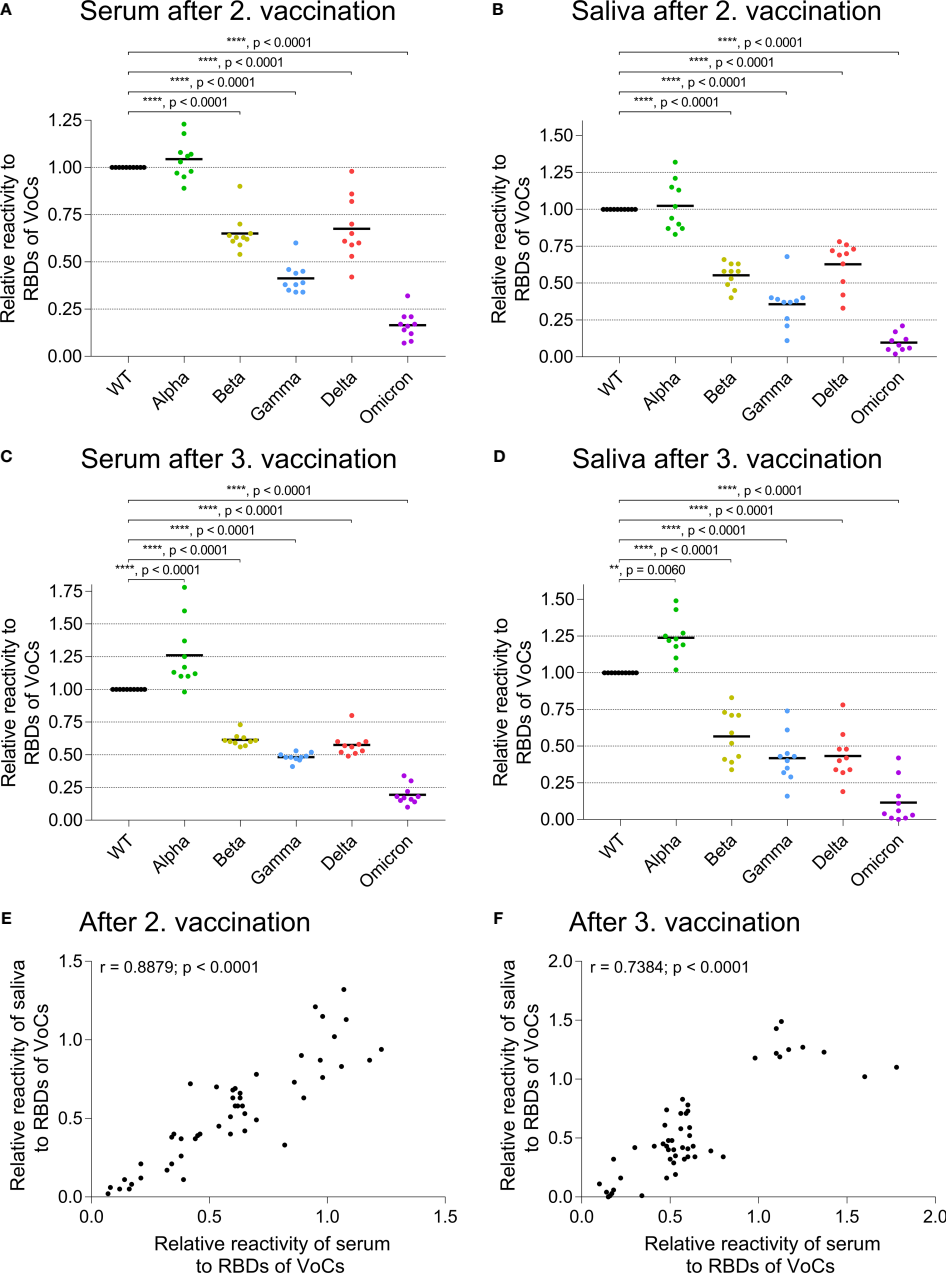
Cross-reactivity of IgG to RBDs of emerging variants in serum and saliva. ELISA plates were coated with RBDs of wild type (WT, black) and of VoCs Alpha/B.1.1.7 (green), Beta/B.1.351 (dark yellow), Gamma/P.1, also called B.1.1.248 (blue), Delta/B.1.617.2 (pink), or the Omicron/B.1.1.529 lineage (violet) SARS-CoV-2 variant. The relative reactivity is shown for each specific response normalized to the reactivity against WT RBD. **(A)** shows the reactivity of serum and **(B)** of saliva two weeks after the second BNT vaccination, **(C)** of serum and **(D)** of saliva two weeks after the third BNT vaccination. Each dot represents one donor. Horizontal lines indicate the mean IgG levels of all donors in the respective groups. The reactivity of the different RBD variants was normalized to the RBD WT (one-way ANOVA, Tukey’s multiple comparison test). **(E, F)** Comparison of SARS-CoV-2 IgG cross-reactivity in serum and saliva. Relative reactivity of specific IgG against RBDs of VoCs reveals a significant correlation between serum and saliva after the second (**E**, Spearman correlation, r = 0.8879, p < 0.0001) and third vaccination (**F**, Spearman correlation, r = 0.7384, p < 0.0001). Each dot represents the response of one donor against an RBD of a VoC as depicted in detail in (**A–D**, each condition n = 10).

We analyzed the cross-reactivity of IgG from serum and saliva samples after the second (**Figures 3A, B**) and the third vaccination (**Figures 3C, D**) against the RBD of five VoCs: Alpha/B.1.1.7, Beta/B.1.351, Gamma/P.1, Delta/B.1.617.2, and Omicron/B.1.1.529 BA.1 (20). The reactivity against the Alpha variant was similar as seen for the WT RBD, whereas the recognition of the Beta and Delta variants was reduced by approximately 30 to 50% (p < 0.0001). The recognition of the Gamma variant was about 50 to 70% lower than that for the WT (p <0.0001), and the Omicron variant was least recognized with a reduction of more than 75% compared to WT (p < 0.0001). This reactivity pattern was similar for serum and saliva after the second and third vaccination with BNT and showed a highly significant correlation as seen in **Figures 3E** (r = 0.8879, p < 0.0001) and **3F** (r = 0.7384, p < 0.0001).

### 3.3 Vaccination induces neutralizing IgG but not IgA in the saliva

**Figure 4:**
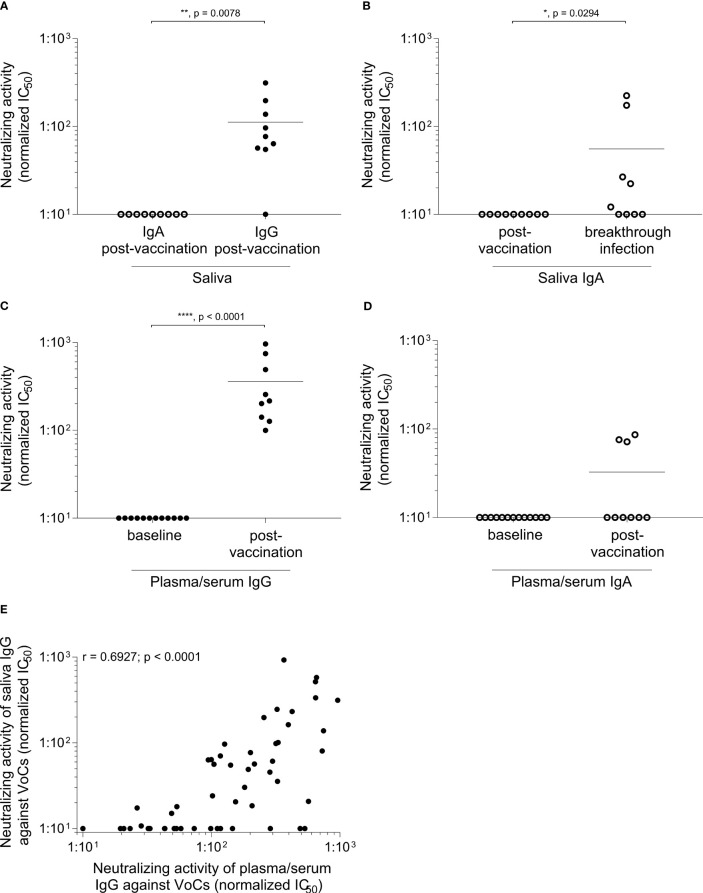
Induction of neutralizing IgG in saliva after vaccination. Samples were collected within one month after the third BNT vaccination. IgG and IgA were purified and a live virus neutralization assay was performed as described in materials and methods. The neutralizing activity is displayed as IC_50_ normalized to 10^7^ viral RNA copies against the EU1/B.1.177 variant of SARS-CoV-2 in **(A–D)** and against the EU1/B.1.177, Alpha/B.1.1.7, Beta/B.1.351, Gamma/P.1/B.1.1.28.1, Delta/B.1.617.2 and Omicron/B.1.1.529 sublineage BA.1 variant of SARS-CoV-2 in **(E)**. In saliva **(A)** only a reactivity is visible for post-vaccination IgG compared to IgA (n = 9; p = 0.0078, Mann-Whitney *U* test). **(B)** After an additional breakthrough infection a clear signal for neutralizing IgA can be obtained (n = 9; p = 0.0294, Mann-Whitney *U* test). Neutralizing activity after IgG and IgA purification of plasma/serum is depicted in **(C, D)**. The baseline IgG (n = 12) and IgA (n = 13) showed no reactivity compared to plasma/serum-derived Ig after vaccination (n = 9) (plasma/serum-derived IgG p < 0.0001, plasma/serum-derived IgA p = 0.0545, Mann-Whitney *U* test). Each dot represents one donor. Closed dots represent IgG and open dots IgA. Horizontal lines indicate the mean IgG levels of all donors in the respective groups. **(E)** Comparison of neutralization of plasma/serum-derived IgG and saliva IgG against different SARS-CoV-2 VoC. Neutralizing IgG reveals a significant correlation between plasma/serum and saliva after the third vaccination (Spearman correlation, r = 0.6927, p < 0.0001). Each dot represents the neutralizing activity of one donor (n = 9) against a SARS-CoV-2 VoC. Detailed responses of the purified IgG from plasma/serum and saliva of each donor to each variant can be found in the **Supplementary Figures 3B, C**.

We further performed a neutralization assay with the authentic SARS-CoV-2 virus variant EU1/B.1.177 (**Figure 4**). When we analyzed the unpurified samples, we could only obtain a neutralizing activity for blood plasma/serum after vaccination but not for the saliva (**Supplementary Figure 3A**). This might be due to the different Ig levels in the material. In serum, the mean IgG level was approximately 7.8 ± 1.8 mg/ml and the mean IgA levels about 1.8 ± 0.6 mg/ml, whereas the mean IgG levels of saliva were about 14.7 ± 13.2 µg/ml and the mean IgA levels about 92.3 ± 64.3 µg/ml.

**Figure 5:**
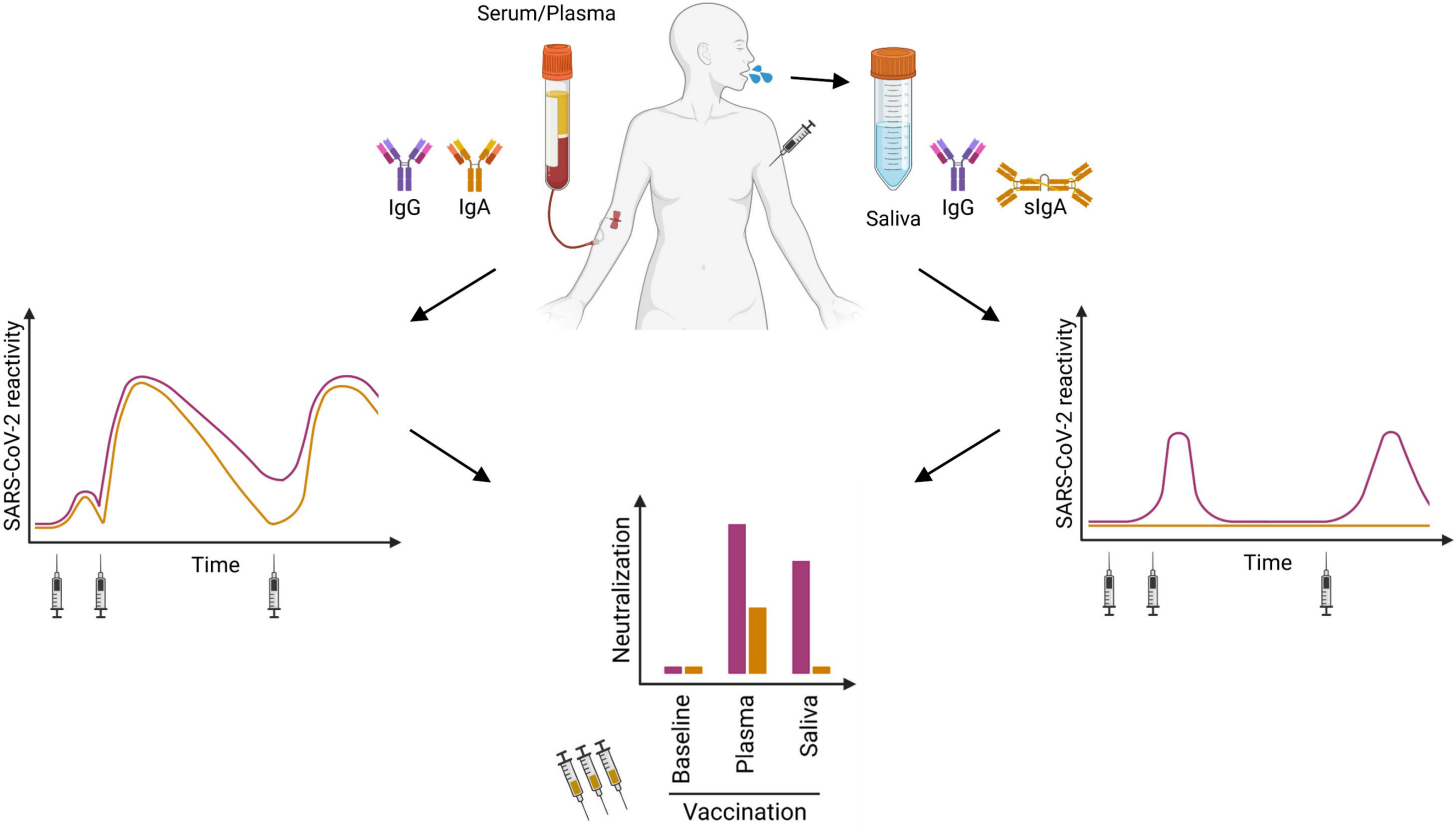
Cartoon summarizing our findings: After the second and third vaccination, SARS-CoV-2-specific IgG and IgA can be found in serum or plasma over six months (left panel). The specific IgG in saliva revealed only a transient reactivity (right panel). After Ig purification, we detected neutralizing IgA in plasma/serum of some vaccinated donors, whereas neutralizing IgG was present within all plasma/serum samples. In saliva from vaccinated individuals, we observed neutralizing activity only in the IgG but not in the IgA fraction (lower panel).

Therefore, we set out to elaborate the functional activity of specific Ig classes, and purified IgG and IgA from blood plasma/serum or saliva. This procedure allowed us to analyze the neutralizing capacity in more detail. Our separation and purification of IgG and IgA from the saliva, has two aspects. First, we can dissect the functional neutralizing activity of IgG versus IgA in the saliva. Second, this increases our sensitivity to detect functional Ig, since both the IgG and IgA concentration in the purified IgG and IgA fractions is higher than in the crude saliva. In the saliva after vaccination (without breakthrough infection), we found neutralizing activity in the IgG, but not in the IgA fraction (**Figures 4A, B**). In contrast, after an additional breakthrough infection, purified IgA from saliva showed a neutralizing activity (**Figure 4B**).

After vaccination, all donors showed a significant increase in neutralizing IgG reactivity (p < 0.0001) in the plasma/serum when compared to baseline IgG from plasma/serum. In contrast, only a few donors harbor neutralizing IgA in their plasma/serum after vaccination (**Figures 4C, D**). Further, we investigated the neutralizing capacity against emerging variants. Here, we could detect a significant correlation between the neutralization of plasma/serum-derived IgG and saliva IgG after the third vaccination against different SARS-CoV-2 VoCs (EU1/B.1.177, Alpha/B.1.1.7, Beta/B.1.351, Gamma/P.1/B.1.1.28.1, Delta/B.1.617.2, Omicron/B.1.1.529 sublineage BA.1, **Figure 4E**, r = 0.6927, p < 0.0001). **Supplementary Figure 3B**, **C** shows the detailed responses of the purified IgG from plasma/serum and saliva of each donor to each variant.

## 4 Discussion

Previous studies analyzing anti-SARS-CoV-2 reactivity in the saliva after vaccination yielded partially contradictory results (14–19). Our study of functional analysis of purified IgA and IgG from the saliva shows that intramuscular vaccination transiently induces a neutralizing activity in the saliva and that this resides in the IgG rather than in the IgA fraction. To analyze features of neutralizing activity against SARS-CoV-2 induced by vaccination, we purified IgG and IgA from saliva and plasma/serum and tested their neutralizing activity against different VoCs. Thereby, we found that vaccination induces a mucosal immunity that is distinct from the mucosal immunity after infection. After vaccination, we found neutralizing IgG, but not neutralizing IgA in the saliva. In serum, however, we detected neutralizing activity from both fractions. We have examined the linkage between total IgG content in serum and saliva with the specific anti-SARS-CoV-2 reactivity in these compartments. As a result, we found a close correlation between serum and saliva IgG reactivity. This is consistent with the view that a fraction of serum IgG is transported to mucosal surfaces via FcRn, while IgA is not transported along this route (11).

Breakthrough infections and primary infections were shown to induce potent IgA in the saliva (4, 5, 25–27). Plasma IgA monomers specific to SARS-CoV-2 proteins were demonstrated to be twofold less potent than IgG equivalents while IgA dimers, the primary form of antibody in the nasopharynx, on average, were 15 times more potent than IgA monomers against the same target (5). Thus, the mucosal immunity after primary infection is distinct from that after vaccination. To achieve great amounts of mucosal IgA after vaccination, mucosal routes of vaccine application are on the horizon (28, 29).

Saliva has been proposed as an alternative for serological studies to detect SARS-CoV-2-specific IgG (30), e.g. in population studies, in particular of children (31). But this approach has to take into consideration that the sensitivity to detect a specific IgG response is higher when serum is analyzed as compared to saliva. This differential sensitivity became clear by our longitudinal analysis over a period of up to more than 16 months. While SARS-CoV-2-specific IgG is readily detected in serum after the first vaccination, two or more vaccinations are needed for the detection of specific IgG in the saliva. The reduced detectability of specific IgG reflects the lower total IgG concentration in the saliva, which was about 500-fold less than serum IgG levels in our study. This concentration difference of IgG between serum and saliva and the lack of transport of serum IgA to saliva (10, 11) explains the reduced sensitivity towards detecting anti-SARS-CoV-2 response in the saliva as compared to serum.

The brief presence of neutralizing mucosal IgG after vaccination might explain why vaccination only transiently induces protection from infection. Our longitudinal ELISA analysis over a period of up to more than 16 months after vaccination showed that the mucosal IgG against SARS-CoV-2 appears only transiently. This aligns with results from previous studies with shorter observation periods (17, 32).

The reduction of IgG after a few months might explain the susceptibility to breakthrough infections, in particular if the waning mucosal immunity is accompanied by the appearance of new VoCs. We found a strong correlation between reactivity to VoCs in serum and saliva. A previous study observed a lack of mucosal reactivity against Omicron, although such a reactivity was seen in serum (17). Limited cross-reactivity to VoCs in the saliva has been described (17, 33). We observed that the cross-reactivity to VoCs in the saliva reflects serum cross-reactivity of IgG, in accordance with the concept that IgG is transported from blood to mucosal sites independent of their antigen-specificity via FcRn (11).

While we did not see neutralizing IgA in the saliva after vaccination, we detected also a systemic IgA response, in accordance with previous observations (34). Our longitudinal ELISA studies indicated a more robust persistence of serum IgG than serum IgA. Six months after the second vaccination, all participants had a specific IgG reactivity in serum while only 71.4% of them had a specific IgA response. This resembles the situation of systemic IgA and IgG after infection, where IgG in serum is more stable than IgA (27), although some individuals loose systemic IgG after infection while they keep memory B cells that can give rise to Abs with neutralizing activity (21). Intriguingly, the circulating IgA may also contribute to the protection after vaccination. It was observed that participants who experienced breakthrough infections with SARS-CoV-2 variants had lower levels of vaccine induced serum anti-Spike/RBD IgA at 2–4 weeks post-dose two compared to participants who did not experience an infection, while total IgG levels were comparable between groups (34).

Further studies are needed to elaborate the clonal relationship between systemic IgA and IgG responses after vaccination against SARS-CoV-2. A recent work analyzing memory B cells and plasmablasts responsive to recall antigens indicated that IgA and IgG memory B cells are clonally related in some cases and might arise from the same germinal centers (35).

We have examined the saliva, but not other mucosal sites as mucosal Abs in nasal secretions are less strongly detected than in the saliva (36). The analysis of nostril swabs, nasopharyngeal aspirate and endotracheal aspirate for SARS-CoV-2-specific Abs has been done and yielded subtle differences between these compartments (37). Furthermore, comparable levels of specific IgG were observed in nasal and oral fluids in a study analyzing Ig in mucosal areas after vaccination (38). The specificity to detect vaccine-induced IgA is reduced, because a few seronegative donors showed an IgA reactivity in saliva but not in serum to SARS-CoV-2. This might be explained by the presence of pre-existing SARS-CoV-2 reactive mucosal B cells in the upper respiratory tract before the pandemic (39). Therefore, we might have missed low levels of IgA recognizing SARS-CoV-2 and we cannot exclude low levels of specific IgA as have been reported (15–17, 34, 40). Importantly, our functional analysis clearly shows that the neutralizing activity after vaccination in the saliva resides in the IgG and not in the IgA fraction.

We have performed our study with persons who were vaccinated with the BioNTech mRNA vaccine BNT162b2. We assume that our observation on the induction of functional IgG rather than IgA in the saliva will also be observed after usage of the Moderna or AstraZeneca vaccine. This expectation is supported by the publications that described reactivity to SARS-CoV-2 in saliva by ELISA (15–17, 34) and the fact that a proportion of IgG in blood is transported to the mucosa via FcRn (12).

In conclusion, our study shows that i.m. vaccination against SARS-CoV-2 induces a transient mucosal immunity, which is characterized by IgG and therefore distinct from the mucosal immunity after infection, which is largely mediated by IgA. The transient neutralizing activity of IgG in the saliva might be indicative of susceptibility to breakthrough infection and may guide recommendations for re-vaccination.

## Data availability statement

The raw data supporting the conclusions of this article will be made available by the authors, without undue reservation.

## Ethics statement

The studies involving humans were approved by Ethikkommission bei der LMU München, Prof. Dr. W. Eisenmenger, Pettenkoferstr. 8a, 80336 München, Germany. The studies were conducted in accordance with the local legislation and institutional requirements. The participants provided their written informed consent to participate in this study.

## Author contributions

SW: Conceptualization, Data curation, Formal analysis, Writing – original draft, Funding acquisition, Investigation, Methodology, Project administration, Resources, Software, Supervision, Validation, Visualization, Writing – review & editing. HR: Formal analysis, Investigation, Methodology, Validation, Writing – review & editing. CÖ: Formal analysis, Investigation, Methodology, Validation, Writing – review & editing. PW: Formal analysis, Investigation, Methodology, Validation, Writing – review & editing. GL: Formal analysis, Investigation, Methodology, Validation, Writing – review & editing. MS: Formal analysis, Investigation, Methodology, Validation, Writing – review & editing. CS: Formal analysis, Investigation, Methodology, Validation, Writing – review & editing. KE: Formal analysis, Investigation, Methodology, Validation, Visualization, Writing – review & editing. SH: Formal analysis, Investigation, Methodology, Validation, Writing – review & editing. HW: Formal analysis, Investigation, Methodology, Validation, Writing – review & editing. DT: Formal analysis, Investigation, Methodology, Validation, Writing – review & editing. MP: Formal analysis, Investigation, Methodology, Validation, Writing – review & editing. MW: Formal analysis, Investigation, Methodology, Validation, Writing – review & editing. PE: Formal analysis, Investigation, Methodology, Validation, Writing – review & editing. BF: Formal analysis, Funding acquisition, Resources, Writing – review & editing. SM: Formal analysis, Supervision, Validation, Visualization, Writing – review & editing. OK: Formal analysis, Funding acquisition, Methodology, Resources, Supervision, Validation, Writing – review & editing, Data curation. TK: Formal analysis, Funding acquisition, Methodology, Resources, Supervision, Validation, Writing – review & editing, Data curation. EM: Conceptualization, Formal analysis, Funding acquisition, Methodology, Project administration, Resources, Software, Supervision, Validation, Visualization, Writing – original draft, Writing – review & editing, Data curation.
