# Supplementary material for: Intramuscular vaccination against SARS-CoV-2 transiently induces neutralizing IgG rather than IgA in the saliva
Source: Front Immunol. 2024 Feb 5;15:1330864. doi: 10.3389/fimmu.2024.1330864 (PMC10875124; doi:10.3389/fimmu.2024.1330864)
Supplement: Supplementary file 1 [file DataSheet_1.pdf]

Supplementary Material

Intramuscular vaccination against SARS-CoV-2 transiently induces neutralizing IgG rather than IgA in the saliva

Stephan Winklmeier[1,2], Heike Rübsamen[1,2], Ceren Özdemir[1,2], Paul R. Wratil[3,4], Gaia Lupoli[3], Marcel Stern[3], Celine Schneider[1,2], Katharina Eisenhut[1,2], Samantha Ho[1,2], Hoi Kiu Wong[1,2], Damla Taskin[1,2], Marvin Petry[1,2], Michael Weigand[5], Peter Eichhorn[5], Bärbel U. Foesel[6], Simone Mader[1,2], Oliver T. Keppler[3,4], Tania Kümpfel[1,2], Edgar Meinl[1,2]*

[1]Institute of Clinical Neuroimmunology, University Hospital, Ludwig-Maximilians-Universität München, Munich, Germany.

[2]Biomedical Center (BMC), Medical Faculty, Ludwig-Maximilians-Universität München, Martinsried, Germany.

[3]Max von Pettenkofer Institute & GeneCenter, Virology, Ludwig-Maximilians-Universität München, Munich, Germany.

[4] German Center for Infection Research (DZIF), partner site Munich, Munich, Germany.

[5]Institute of Laboratory Medicine, University Hospital, Ludwig-Maximilians-Universität München, Munich, Germany.

[6]Institute of Epidemiology, Helmholtz Munich, Neuherberg, Germany.

*** Correspondence:**
Dr Edgar Meinl
edgar.meinl@med.uni-muenchen.de

1 Supplementary Figures

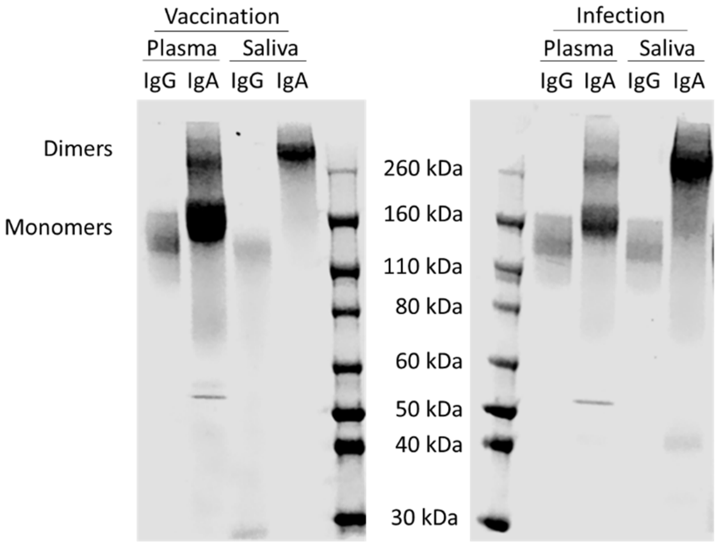

Supplementary Figure 1. Analysis of monomeric or dimeric IgA and IgG in blood and saliva. Plasma- and saliva-derived and purified IgG and IgA (obtained after the third vaccination (left) or after an additional breakthrough infection (right)) were separated by non-reducing SDS-PAGE gel and stained by Coomassie Blue.

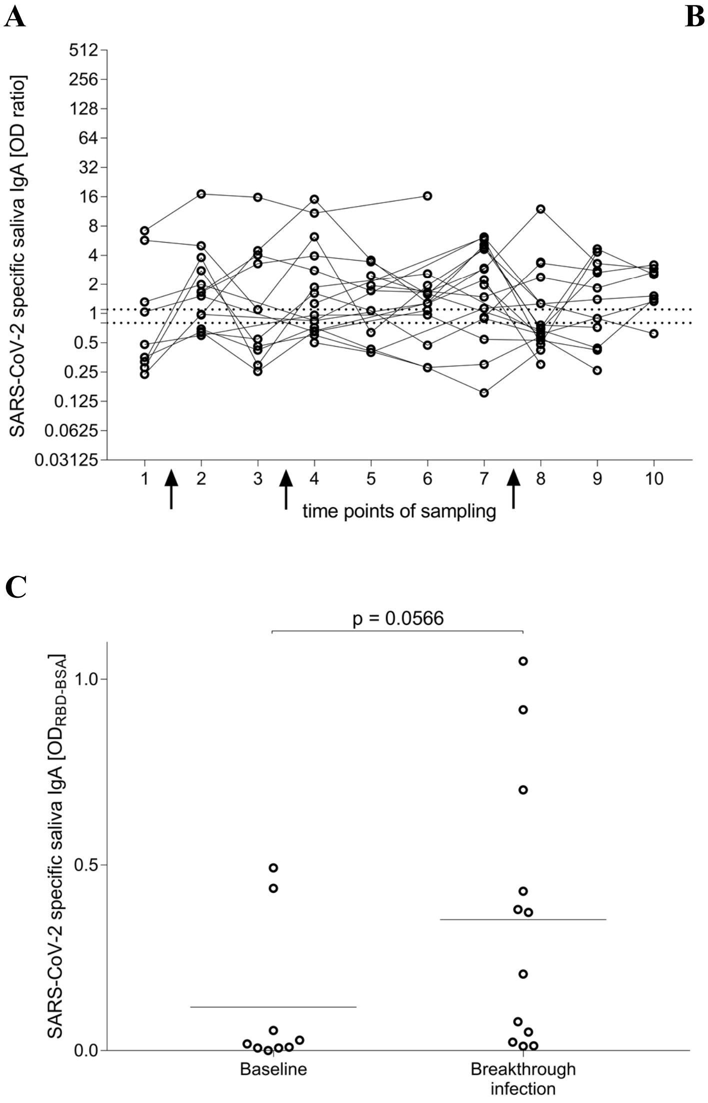

Supplementary Figure 2. Analysis of SARS-CoV-2 specific IgA in the saliva by ELISA. IgA response to the S1 domain or RBD WT of the SARS-CoV-2 spike protein after BNT vaccination or breakthrough infection obtained by the EUROIMMUN ELISA kit (**A**) or an in-house ELISA following a previous publication (1) (**B** and **C**). (**A** and **B**) Longitudinal reactivity of specific IgA in saliva. Arrows indicate vaccination time points one, two and three. Connection between dots refer to the response within one donor. Time points of sampling: 1 = before first vaccination (n_A = 9, n_B = 9); 2 = two weeks after first vaccination (n_A = 14, n_B = 14); 3 = before second vaccination (n_A = 10, n_B = 10); 4 = two weeks after second vaccination (n_A = 16, n_B = 11); 5 = six weeks after second vaccination (n_A = 9, n_B = 9); 6 = three months after second vaccination (n_A = 13, n_B = 13); 7 = six months after second vaccination (n_A = 17, n_B = 13); 8 = two weeks after third vaccination (n_A = 17, n_B = 12); 9 = three months after third vaccination (n_A = 12, n_B = 9); 10 = six months after third vaccination (n_A = 8, n_B = 8). The area between the two horizontal dotted lines in (**A**) were considered to represent the borderline zone of reactivity (EUROIMMUN). For comparison, we included these lines for serum (**Figure 1C**) also here in the graph of saliva IgA. (**C**) Detection of SARS-CoV-2 RBD-specific IgA in saliva from baseline (n = 9) and after breakthrough infection (n = 12) from three times vaccinated participants. Horizontal lines indicate the mean levels of all donors in the respective groups (p = 0.0566, Mann-Whitney *U* test).

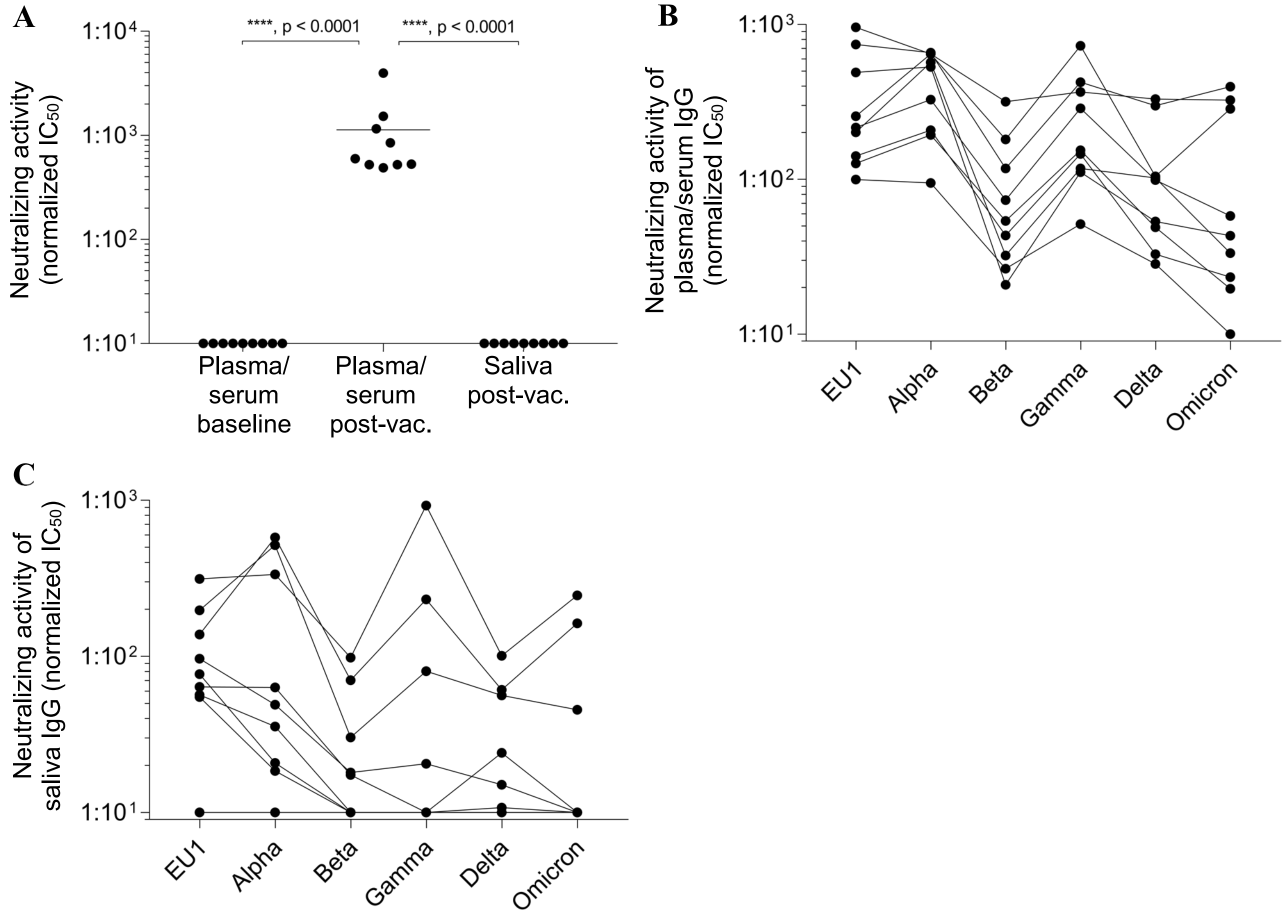

Supplementary Figure 3. Analysis of neutralizing activity against variants of concern of SARS-CoV-2. Neutralizing activity before (**A**) and after the third vaccination (post-vac.; **A**, **B** and **C**). Samples were collected within one month after the third BNT vaccination. S3 neutralization assay was performed as described in materials and methods. (**A**) The neutralizing activity is displayed as IC_{50} normalized to 10^7 viral RNA copies against the EU1/ B.1.177 variant of SARS-CoV-2. The neutralizing activity before Ig purification is shown for baseline plasma/serum, plasma/serum and saliva after the third vaccination ($n = 9$; $p < 0.0001$, one-way ANOVA, Tukey's multiple comparison test). Each dot represents one donor. Horizontal lines indicate the mean levels of all donors in the respective groups. (**B**) depicts the neutralizing activity of plasma/serum-derived IgG ($n = 9$) and (**C**) of saliva-derived IgG ($n = 9$) after the third vaccination against different SARS-CoV-2 VoCs (EU1/B.1.177, Alpha/B.1.1.7, Beta/B.1.351, Gamma/P.1/B.1.1.28.1, Delta/B.1.617.2, Omicron/B.1.1.529 sublineage BA.1).

2 References

1. Darwich A, Pozzi C, Fornasa G, Lizier M, Azzolini E, Spadoni I, et al. BNT162b2 vaccine induces antibody release in saliva: a possible role for mucosal viral protection? EMBO Mol Med. 2022;14(5):e15326.
